# *In silico *characterization of microbial electrosynthesis for metabolic engineering of biochemicals

**DOI:** 10.1186/1475-2859-10-76

**Published:** 2011-10-03

**Authors:** Aditya V Pandit, Radhakrishnan Mahadevan

**Affiliations:** 1 Department of Chemical Engineering and Applied Chemistry, University of Toronto, 200 College Street, Toronto, M5S 3E5 Canada; 2 Department of Chemical Engineering and Applied Chemistry, University of Toronto, 200 College Street, Toronto, M5S 3E5 Canada; Institute of Biomaterials and Biomedical Engineering, University of Toronto, 164 College Street, Toronto, M5T 1P7 Canada

## Abstract

**Background:**

A critical concern in metabolic engineering is the need to balance the demand and supply of redox intermediates such as NADH. Bioelectrochemical techniques offer a novel and promising method to alleviate redox imbalances during the synthesis of biochemicals and biofuels. Broadly, these techniques reduce intracellular NAD^+ ^to NADH and therefore manipulate the cell's redox balance. The cellular response to such redox changes and the additional reducing power available to the cell can be harnessed to produce desired metabolites. In the context of microbial fermentation, these bioelectrochemical techniques can be used to improve product yields and/or productivity.

**Results:**

We have developed a method to characterize the role of bioelectrosynthesis in chemical production using the genome-scale metabolic model of *E. coli*. The results in this paper elucidate the role of bioelectrosynthesis and its impact on biomass growth, cellular ATP yields and biochemical production. The results also suggest that strain design strategies can change for fermentation processes that employ microbial electrosynthesis and suggest that dynamic operating strategies lead to maximizing productivity.

**Conclusions:**

The results in this paper provide a systematic understanding of the benefits and limitations of bioelectrochemical techniques for biochemical production and highlight how electrical enhancement can impact cellular metabolism and biochemical production.

## Background

In response to economic and environmental considerations, there has been increased interest in the development of commercial bioprocesses that produce biofuels and specialty chemicals. The successful commercialization of these bioprocesses requires that the bioproducts which are converted from biomass based feedstocks are produced at sufficiently high yields and productivity so that the process is economically viable [1]. Hence, engineering the metabolism of organisms that drive these fermentative processes, such as *Escherichia coli *and *Saccharomyces cerevisiae*, is required to achieve desired process objectives (product yield, productivity, and titer) [2-4]. Metabolic engineering attempts to optimize the cellular metabolism of an organism to satisfy these desired process objectives. Typically, this is achieved by introducing exogenous metabolic pathways and manipulating native metabolic pathways, or by manipulating cellular redox and energy reactions in order to overproduce desired metabolites [3,5].

Because redox cofactors such as NADH or NADPH play an important role in cellular metabolism, altering the cellular redox balance has been regarded as an essential initial step in metabolic engineering for achieving bioprocess objectives [6]. Genetic manipulation of various enzymatic pathways offer means to increase the NAD(P)H available to the cell [7] and has been demonstrated as an effective way to increase the synthesis of desired products [8-10]. An alternative approach for manipulating the redox metabolism is the use of bioelectrochemical techniques such as those that supply reducing power by generating reduced NADH within the cell through interactions with an electrode. These techniques have been shown to be effective at increasing synthesis of several products including ethanol, *n*-butanol, and succinate in a variety of hosts including *S. cerevisiae, Clostridium acetobutylicum, Actinobacillus succinogenes *[11-13] Other microbes have also been successfully utilized for biotransformations or product synthesis [14,15].

These types of bioelectrochemical techniques, which refer to electricity driven product synthesis, are generally known as microbial electrosynthesis or bioelectrosynthesis [16]. In 1979, Hongo *et al*. were among the first to use these techniques to increase product synthesis by showing that it was possible to improve L-glutamic acid yields [17]. Considerable progress has been made since 1979 in the area of bioelectrosynthesis. Reduced carriers such as neutral red and methyl viologen have been shown to increase the yields of products such as ethanol, butanol and succinate and direct electron transfer has also been demonstrated in mediator free bioelectrochemical systems where electron transfer has occurred between the cell membrane and the cathode [16]. In 2004, it was demonstrated that an electrode could serve as a sole energy source for *Geobacter sulfurreducens *[18]. Recently, Lovley *et al*. demonstrated a proof of concept for this approach; *Sporomusa ovata *was used to generate acetate and small amounts of organic compounds by reducing carbon dioxide with an electrode powered by solar energy [19]. However, practical industrial implementation would require further genetic perturbations to the central metabolic pathways of *S. ovata *to develop strains that could produce valuable chemicals instead of acetate.

The identification of genetic perturbations to a metabolic network has been aided by *in silico *genome scale models and computational algorithms such as Opt-Knock, OptForce and EMILiO that identify knockout, overexpression and/or inhibition strategies [20-22]. Currently, however, these computational tools have been limited to perturbations that affect the metabolism through genetic manipulation techniques and not through electrochemical techniques. The lack of similar computational tools that could aid in the understanding of electrochemical perturbations to the cellular metabolism and the growing interest in microbial electrosynthesis motivates the development of a computational framework that can be used in the rational design of strains.

The lack of such a framework means that there is also a lack of systematic understanding of the instances where bioelectrosynthesis can lead to improved product yield (electrical enhancement). Hence, in order to characterize the role of electrical enhancement on chemical production, in this study we (1) develop a method to analyze the impact of microbial electrosynthesis on biochemical production using the genome-scale metabolism of *E. coli *as an example; (2) examine the role of microbial electrosynthesis on biomass growth; (3) examine the impact on biochemical production for a suite of top value-added chemicals; (4) identify which conditions microbial electrosynthesis is best suited as a tool to improve yield; and (5) comprehensively evaluate how microbial electrosynthesis may impact strain design and process productivity. These results provide valuable insights on the role of microbial electrosynthesis and highlight the need for additional studies to optimize this process.

## Methods

### 2.1 Modelling Bioelectrosynthesis for Chemical Production

The iAF1260 metabolic reconstruction of *Escherichia coli *was used as the basis for all *in silico *evaluations [23]. The iAF1260 model was selected because it is well-curated, studied and experimentally validated. Moreover, genetic tools for *E. coli *are well established and it is used widely in industry as platform for biochemical production. Even though *E. coli *is not a natural electricigen in the way that *Geobacter *or *Shewanella *are, *E. coli *can be electroactive and interact with an electrode in the presence of mediators [24,25]

Recently *E. coli *was shown to reduce solid α-Fe_2_O_3 _in the absence of mediators after portions of the extracellular electron transfer chain of *Shewanella oneidensis *was expressed in *E. coli *[26]. This ability to reduce metals is a trait that mimics natural electricigens such as *Geobacter sulfurreducens*, suggesting that direct electron transfer between electrodes may be a possibility.

### 2.2 Modelling Electrode Interactions

**Figure 1:**
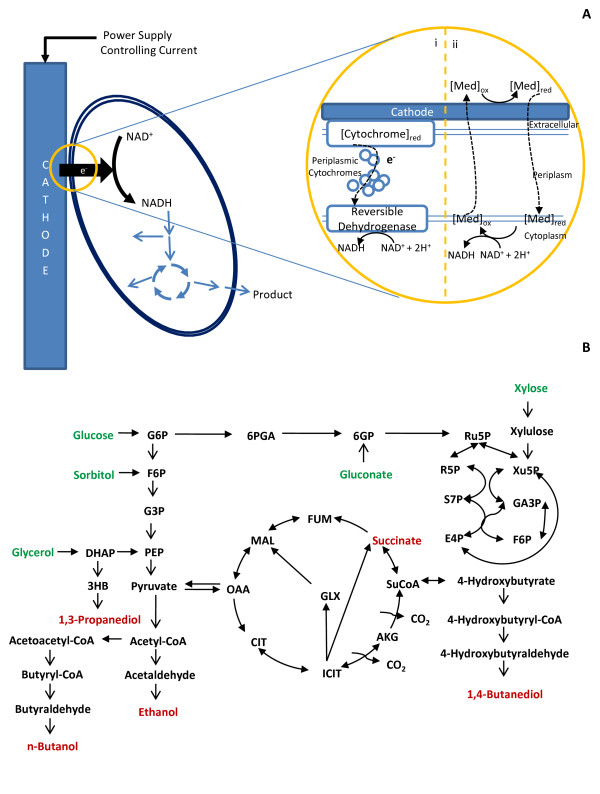
**Central Metabolism and NADH Regeneration Maps**. (A) A schematic of the bioelectrosynthesis process. (i) shows proteins that would likely be necessary for reverse electron flow to drive bioelectrosynthesis. (ii) Shows bioelectrosynthesis by a mediator driven process. (B) The central metabolism of *E. coli *shown with the products that were analysed in this study. The key abbreviations are provided in the supplemental information.

To account for the interactions with an electrode, two reactions were added to the model reconstruction. These reactions are described in Additional file 1 and represent the net reaction that occurs between the electrode and free NAD^+ ^in the cytoplasm through the quinone pool. They are based on pathways used by bacteria such as *Acidithiobacillus ferrooxidans*, whose outer membrane has cytochromes that are responsible for oxidizing iron ore [27-29]. Figure 1A shows this process schematically. While these proteins are not native, *E. coli *is known to interact with an electrode when neutral red serves as a mediator, and this interaction has been demonstrated to affect its cellular metabolism [30]. Furthermore, *E. coli *has shown dissimilatory iron reduction under cymA expression from *Shewanella oneidensis *[31].

Figure 1A shows this process schematically. The lower value for the electron uptake rate was set based on known and measurable reverse electron flows that occur in electricigens. Bond and Lovley measured the electron donation rate to an electrode for *G. sulfurreducens *at 1.2 μmol/mgProtein-min or 1.07 A/gDW [32]. The same study reported a corresponding current production at 1.143 A/m^2^. Xie *et al *showed that *E. coli *is capable of becoming redox active in the presence of an electrode modulated by a computerized potentiostat [25]. Based on their data, an electron donation rate of *ca*. 600 mA/gDW was calculated. The electron uptake rate used in this study (*ca*. 800 mA/gDW) is consistent with these reported values.

The calculation of growth rates and product flux was computationally determined using a Flux Balance Analysis (FBA) framework. The FBA framework has been previously used to analyze growth of *E. coli *[33,34]. Computations were performed in MATLAB (The Math-Works Inc., Natick, MA, USA) and the COBRA Toolbox [35].

In addition to the electron transfer reactions described earlier, a number of other modifications were made to the model to account for growth-coupled product flux and the production of biochemicals via pathways not native to *E. coli*. These heterologous pathways had been previously identified in literature and were incorporated into the model [36-40]. A summary of these reactions and the corresponding growth coupled knockouts is provided in the additional files (See Additional file 1).

Unless specifically noted, all simulations were performed under anaerobic conditions. A substrate uptake rate of 10 mmol/gDW-hr for glucose was used. For ease of comparison to other substrates on an equal carbon basis, the following values were used for other substrates: 10 mmol/gDW-hr for gluconate and sorbitol. 12 mmol/gDW-hr for xylose; 20 mmol/gDW-hr for glycerol and 5 mmol/gDW-hr for maltose. The electron uptake rate was limited to a maximum of 30 mmol/gDW-hr. The model was allowed to choose any uptake rate that maximized the product flux (or biomass) between 0 and 30 mmol/gDW-hr.

### 2.3 Selection of Substrates and Products for Analysis

*E. coli *is capable of growing on a number of different carbon sources. The carbon source utilized by the organism can impact the distribution of the fermentation products during anaerobic growth. The change in the by-product secretion patterns, and the corresponding yield of a product, is often associated with the degree of reduction (defined below) of the substrate and the external redox state. Electrical enhancement introduces another perturbation to the cell's redox state. Therefore, to characterize the general impact of electrical enhancement on product yield, we considered various substrate-product pairs, and modelled growth under a variety of different carbon substrates. Many of these product compounds have been identified as commercially valuable by the *US Department of Energy *[41]. Table 1 shows the list of carbon sources that were selected as substrates and products.

**Table 1:** NADH Produced or Consumed per Substrate or Product

Products			Substrates		
	**NADH Consumed**	**NADH/# of C**		**NADH Produced**	**NADH/# of C**

Succinate	2	0.5	Glucose	2	0.33

1,3-Propanediol	3	1.0	Xylose	1.67	0.33

1,4-Butanediol	6	1.5	Glycerol	2	0.67

*n-Butanol*	4	1.0	Maltose	4	0.33

Ethanol	2	1.0	Sorbitol	3	0.50

			Gluconate	1	0.17

To establish a systematic method to analyze the relationship between the substrate-product coupling, we compared the yield improvements as a function of the *degree of reduction *of the substrate and product. We defined the degree of reduction as the number of reducing equivalents produced by a substrate during its oxidation to pyruvate or the number of reducing equivalents consumed to produce a desired metabolite from pyruvate, divided by the carbon length of that substrate or product. These values are shown in Table 1. We then divided the degree of reduction of the substrate by that of the product to create single parameter which we defined as the Substrate Product Electron Equivalence Quotient (SPEEQ). We related this parameter to product yield improvement. SPEEQ provides information on the relative degree of reduction of substrate to the product, and if SPEEQ is greater than one, it implies that the substrate is more reduced than the product and vice-versa.

The products specified in Table 1 were carefully selected so that we could test the effect of the products' degree of reduction on the yield improvement. For example, products such as ethanol and *n*-butanol allowed us to test the effect of keeping the degree of reduction constant while varying the carbon length and pathway dependency, while products such as succinate, 1,3-propanediol and 1,4-butanediol allowed us to evaluate how different degrees of reduction impact yield. Figure 1B shows the pathways present in the central metabolism of *E. coli *that consume or produce these substrates and products.

## Results and Discussion

### 3.1 Impact on ATP Yield and Biomass

**Figure 2:**
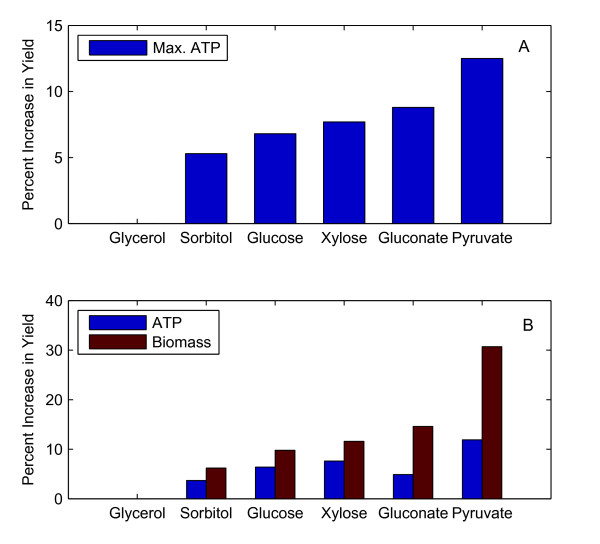
**Percentage Improvements in ATP Yield and Biomass Yield as a Result of Electrical Enhancement**. (A) The improvement in the theoretically maximum synthesis of ATP by electrical enhancement. (B) The improvement in biomass growth rate under electrically enhanced conditions.

Characterizing changes to ATP yield and biomass growth rate are fundamental to understanding how electrical enhancement impacts cellular metabolism. To determine the extent to which electrical enhancement can influence ATP production, we calculated the maximal ATP yield that could be generated in the presence of electrical enhancement. While the maximization of ATP (without biomass) is not perhaps a physiologically valid objective function, it serves two purposes in understanding how bioelectrosynthesis influences the energetics of the underlying metabolic network. Firstly, it helps to isolate and identify the energy producing pathways that could make the largest contribution to additional ATP for the cell. Secondly, comparison of relative increases in maximal ATP production against increases in biomass yield can help distinguish whether the biomass production is limited by ATP, redox, or carbon availability. By analyzing the differences in ATP and biomass yields, it is possible to identify the constraints on the metabolic network for increasing ATP and biomass yields by bioelectrosynthesis. The results of the simulations are shown in Figure 2A.

Figure 2A shows electrical enhancement is capable of increasing the ATP yield in range of 5-9% of the base case (without electrical enhancement). More importantly, the predicted **absolute **increase in the ATP produced was identical, 1.9 mmol/gDW-hr in all cases except for sorbitol and glycerol utilizing conditions. These two substrates are exceptions because they are already highly reduced and the extra electrons supplied during bioelectrosynthesis have a lower or no benefit on ATP production. The results suggest two important points. The increase in ATP produced by the cell during electrical enhancement is directly proportional to the current supplied to the cell. Secondly, the mechanism by which ATP is produced is linked to specific metabolic pathways that are described in detail in the following section.

Since there are a limited number of reactions in the central metabolism that are capable of generating ATP under anaerobic conditions, (acetate kinase (ACKr), pyruvate kinase (PYK), phosphoglycerate kinase (PGK), ATP synthase (ATPs4rpp)), analysis of these four pathways can suggest a mechanism by which electrical enhancement improves ATP yields (Figure 2A).

Physiologically, *E. coli *operates ATP synthase as a low flux ATP driven proton pump under anaerobic conditions [42]. This pump discharges protons during anaerobic growth to generate an electrochemical proton gradient usually required for solute transport and flagellar rotation. During bioelectrosynthesis, electrons are transferred into the cell. In order to maintain charge neutrality, the addition of electrons must be accompanied by the transfer of protons into the cytoplasm. One of the main sources of protons for the cell is ATP synthase which is driven by a proton gradient. For every four moles of protons transported into the cell, ATP synthase also produces one mole of ATP. Hence, the addition of electrons can directly enhance ATP generation through the reversal of ATP synthase from ATP consumption (proton efflux) to ATP production (proton influx). Details of the changes in the flux distribution can be found in Additional file 2.

This analysis seems to indicate that energy yields within the cell could be manipulated by controlling the current of the electrode, since an increased flux through the NADH electrode generation reaction could facilitate the generation of a proton gradient. The ability to increase ATP yields within a cell offers new perspectives on how strains could be designed to maximize growth rates, particularly under fermentative conditions when cells are usually energy starved. We examined the ATP production when the objective function was assumed to be maximization of growth rates *in silico *to further test this idea.

The change in the net ATP flux produced by the four pathways (kinases and the ATP synthase) available to *E. coli *under anaerobic conditions (biomass objective) was calculated and plotted as a percentage of the base case (no electrical enhancement). Figure 2B shows that increases in biomass yield from electrical enhancement do occur and that the enhancements, with the exception of pyruvate and glycerol utilizing conditions, fall in a limited range.

Figures 2A and 2B together help elucidate the role that generation of NADH by bioelectrosynthesis has on biomass yields through the manipulation of both cellular energetics (ATP) and cellular redox conditions (NADH). Consider that for glucose and xylose utilizing conditions, there is an expected increase of 6.3% and 7.6% in ATP yield, and 10% and 12% in biomass yield, respectively. By comparison, when gluconate is used as the substrate, the increase in ATP and biomass yield is 4.9% and 15%, respectively. In contrast, a significant disparity between increases in ATP yield and biomass yield can be observed for pyruvate (12% and 31% increase in ATP & biomass respectively). These results suggest that the improvements in yield are not the sole result of additional ATP generated by a proton motive force (described above). For example, if increases in biomass yield arose only from ATP, then the increase in biomass yield on pyruvate predicted by the simulation should be less marked. Rather, it is the combination of reducing power available to the cell (dependent on current supply at the electrode and the substrate's degree of reduction), *and *ATP that leads to higher growth yields. This result suggests that relative increases in biomass yields are greater for oxidized substrates than for more reduced ones.

The above conclusion has the following implications for industrial bioelectrosynthesis. In the presence of an NADH drain on the system, such as biosynthetic pathways, or pathways that might be required for the production of highly reduced metabolites, bioelectrosynthesis offers the possibility of boosting biomass yields by supplying reducing power as well as producing a proton motive force that could generate ATP. This concept is particularly important for strain designs that result in poor growth rates. These strains are usually not industrially viable because of their poor growth rates, but could be made viable by electrically boosting growth rates while maintaining specific knockout strategies geared towards metabolite production. Interestingly, though perhaps by a different mechanism, Park and Zeikus found that electrical enhancement with neutral red as an electron mediator for *A. succinogenes *was able to drive proton translocation and increase ATP synthesis [13]. Their experimental results showed that the strain undergoing bioelectrosynthesis consumed significantly more glucose and had greater biomass and succinate production at the end of the batch.

Analysis of the flux distributions of the central metabolism provide insight into how these improvements can be achieved. The wild-type metabolic network of *E. coli *shows increases in flux through parts of the pentose phosphate pathway, mid glycolysis and the branched TCA cycle. Changes to the flux of the reactions that belong to these pathways are generally similar and are approximately 10% of the base case. Significant changes in flux appear in those reactions that are capable of consuming NADH. These changes include: 1) the pyruvate metabolism for which ethanol producing pathways have higher fluxes (90% increase; 2) the acetate producing pathways which have a lower flux value (90% decrease); and 3) ATP synthase shows a change in directionality (for reasons previously described). A metabolic map describing the changes to fluxes in the central metabolism is provided in the Additional file 3.

### 3.2 Impact on CO_2 _Fixing Pathways

Bioelectrosynthesis provides reducing power and if used in conjunction with highly oxidized carbon sources such CO_2_, it may be possible to substantially improve yields [43]. We explored this concept further by incorporating the Wood-Ljungdahl pathway into the model [44] (See Additional file 2).

The results shown in Table 2 suggest that simultaneous CO_2 _fixation and electrical generation of NADH can lead to a substantial improvement in biomass yield and growth rate. The improvement in growth rate is almost two fold relative to the wild type under electrically enhanced and carbon fixing conditions. The Wood-Ljungdahl pathway produces acetyl-CoA as the final product. A fraction of this can be converted to acetate to generate ATP while part of the acetyl-CoA can be transformed to biomass precursors provided that NADH cofactor requirements for these reactions are met. Electrical enhancement provides some of the reducing power that is necessary to meet these requirements. Without enhancement, glucose is the sole supply of reducing power leading to lower relative yields.

**Table 2:** Effect of CO_2 _Fixation on Growth Rate

Condition	Wild Type (Glucose)	Glucose with Enhancement	Glucose and Wood- Ljungdahl Pathway	Glucose and Wood- Ljungdahl Pathway with Enhancement	Enhancementon only CO_2 _
Growth Rate (hr^-1^)	0.19	0.21	0.31	0.38*	0.008

Growth Yield (×10^2^)**	3.1	3.4	5.2	5.8	--

*Carbon from CO_2 _represented roughly 10% of total carbon. Carbon from CO_2 _as a faction of total carbon (glucose + CO_2_) was negligible for other conditions.

** Defined as Growth Rate/(6*Total Incoming Glucose + Total Incoming Carbon Dioxide)

The growth yield shows the total biomass produced relative to total incoming carbon is larger with electrical enhancement than without. This increase occurs due to a combination of additional CO_2 _fixed and less carbon secreted as metabolites such as formate. Therefore, bioelectrosynthesis improves the specificity of the biomass reaction by incorporating more carbon into biomass precursors. While carbon fixation can be used to increase biomass and improve product yields in the presence of glucose (or some other substrate), we wanted to evaluate the potential for bioelectrosynthesis when CO_2 _is the sole carbon source. The maximum theoretical succinate flux was used as the criteria for evaluating this potential. The maximum theoretical succinate production was calculated to be 0.38 mmol/gDW-hr at a maximum electron uptake rate of 30 mmol/gDW-hr. The calculated succinate flux is very low and this suggests that much larger electron uptake rates are required to achieve product fluxes that are comparable to succinate production from glucose.

The results seem to suggest that the best strategy, in the short term, for synthesizing chemicals or fuels would be the co-utilization of CO_2 _with other hexose and pentose sugars. Our data suggests the microbes capable of reducing CO_2 _to a desired metabolite such as succinate would require an electron uptake flux an order of magnitude larger than what we used in the model. The equivalent electron flux for substituting glucose would be 2.4 × 10^2 ^mmol/gDW-hr for 10 mmol/gDW-hr of glucose. This flux corresponds to current density of 6.8 A/m^2 ^assuming biofilm cell densities reported in the literature [32]. In some cases it may be possible to supply a current sufficient for bioelectrosynthesis to microbes, because lower rates may be required for synthesizing less reduced metabolites. However, using current microbial fuel cell technology as a basis for drawing some conclusions, it becomes apparent that achieving sufficiently high current exchange rates with an electrode can be difficult in non-electricigens since one of the highest reported electron transfer rates for electricigens in microbial fuel cells (even though this transfer is in the direction of current generation) is 7.6 A/m^2 ^[45]. This potential limitation in supply of electrons also suggests avenues for future research to improve the electron transfer rate through a better understanding of the microbe-electrode interactions particularly when the electrode is used as the donor [46]. The results are significant because they help put into perspective some of the challenges that will need to be overcome in order for microbial electrosynthesis to use CO_2 _as the sole carbon source.

### 3.3 Impact of Product and Substrate Degree of Reduction on Bioelectrosynthesis

Product yield improvements due to electrical enhancement are dependent on the biochemical compound produced and the substrate utilized. We computed the maximum theoretical product flux for each biochemical compound of interest from each of the substrates. The maximum product yield occurs under conditions of no biomass synthesis and can be computationally determined by solving the stoichiometric model and maximizing the product exchange flux rather than the biomass growth reaction. Maximizing for product synthesis redirects carbon flux and therefore electron transfer to product formation rather than biomass synthesis and establishes the upper limit on the product yield.

Changes to the theoretical maximum product flux under electrical enhancement provides insight into the relationship between the substrate and the product, and helps to identify the substrate product electron equivalence quotient (SPEEQ) values for which electrical enhancement is most useful.

**Figure 3:**
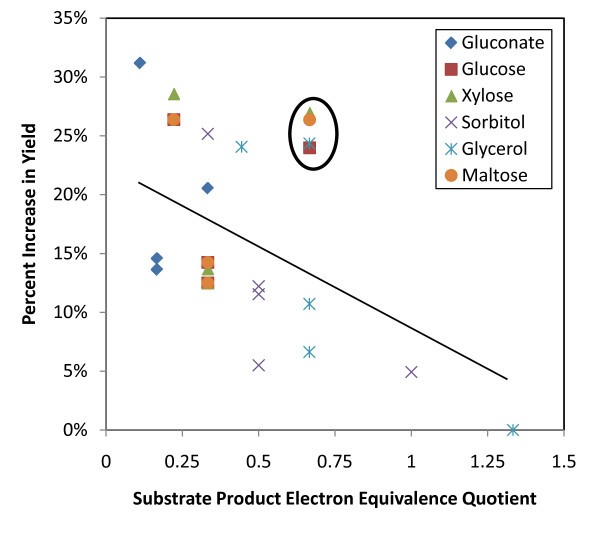
**Theoretical Increases in Product Yield Resulting from Electrical Enhancement**. Theoretical improvement in product yield resulting from electrical enhancement using the wild type metabolic network. Substrate Product Electron Equivalence Quotient Represents the various product-substrate combinations.

There are two discernible conclusions in Figure 3. The first is the expected trend that shows the achievable product yield improvement is generally dependent on the SPEEQ leading to the conclusion that when the reducing power of the substrate is high relative to the product's low degree of reduction, electrical enhancement is of little significance. The converse of this also appears true; electrical enhancement is much more significant in improving yields when the reducing power of the substrate is relatively small compared to the product's degree of reduction.

While these results are along expected lines, there are a few data points that cluster above the trending line (circled), which suggest large improvements in yield are possible even when the substrate product electron equivalence quotient (SPEEQ) is relatively high. Analysis shows that these points are associated with the production of succinate and 1,4-butanediol, and can be attributed to the CO_2 _reducing pathways present in the *E. coli *metabolism. Phosphoenolpyruvate carboxylase (*ppc*) is the enzyme responsible for this function. Consequently, the additional NADH generated by reactions at the electrode induces the cell to shift its metabolism towards pathways that balance the redox allowing the incorporation of carbon dioxide. This results in a substantial improvement in yield. Hence, this provides an explanation for the counter intuitive observation that large improvements are possible even when SPEEQ is high. It should be noted however, recently, yield improvements may be possible for high SPEEQ conditions when an electrode serves as an electrode sink rather than an electron donor. Flynn *et al*. were able to show that *Shewanella oneidensis *could be used to ferment glycerol to ethanol in the presence of an electrode based electron acceptor [47].

A closer look at the output from the simulations reveals the importance that *ppc *activity has on yield improvement. For succinate production, the flux through this reaction can be increased by as much as 14-44% for optimum production under electrically enhanced conditions. The relative increase in 1,4-butanediol production is even greater when the *ppc *activity is present. The predicted flux of *n*-butanol is also increased under electrically enhanced conditions, however this is attributable largely to the additional reducing power available to drive the reactions forward. The flux through *ppc *is relatively low for *n*-butanol synthesis compared to other products since acetyl-CoA is the precursor metabolite and not oxaloacetate. Fluxes for a few key reactions are shown in Table 3.

**Table 3:** Predicted Fluxes Through Selected Reactions

	Succinate		1,4-Butanediol		Ethanol		*n*-Butanol	
Reaction	Without EE	With EE	Without EE	With EE	Without EE	With EE	Without EE	With EE
*ATPS4rpp*	-1.8	5.3	2.7	11.7	-11.6	13.1	-11.6	-1.6
*ppc*	13.7	18.4	8.6	10	0	13.6	0	2.5
*ackr*	-3.9	-1.4	2.1	6.8	0	0	0	0

*Units: mmol/gDW-hr

While studies have shown that overexpression of *ppc *has resulted in higher succinate production under anaerobic conditions [48] this data suggests that electrical enhancement could serve as a means to regulate the flux through *ppc *as a response to NADH generation. Increasing the applied current to the cells would result in increased NADH which would subsequently change the cell's NADH/NAD^+ ^ratio. Those pathways that are capable of regenerating NAD^+ ^would in turn see higher fluxes. When these pathways incorporate CO_2_, the product yields are relatively higher. This result suggests that as other pathways of carbon fixation that regenerate NAD^+ ^are incorporated into the metabolism, their fluxes may be controlled by directly influencing the NADH generation rate through an electrode.

Theoretical improvements in yield are notably similar for products that have the same degree of reduction but are metabolically many reaction steps apart. Ethanol and *n*-butanol are examples of these and they exhibit similar enhancement. Additionally substrates with the same degree of reduction (glucose, maltose, xylose) show almost identical levels of enhancement once again suggesting the importance that the SPEEQ has on product yield improvement.

### 3.4 Growth Coupled Electrical Enhancement

Under growth coupled product formation, electrical enhancement can impact the metabolism in a number of ways. We evaluated two scenarios where electrical enhancement could significantly impact the cell, and therefore, the growth coupled product flux. We examined the impact of electrical enhancement on (1) growth coupled strategies for various substrate-product couplings to show that electrical enhancement can be used on under growth coupled scenarios (see Additional files 1, 4 & literature), and (2) strain design strategies and changes to their corresponding substrate specific productivity under electrically enhanced conditions.

#### 3.4.1 Growth Coupled Strategies

**Figure 4:**
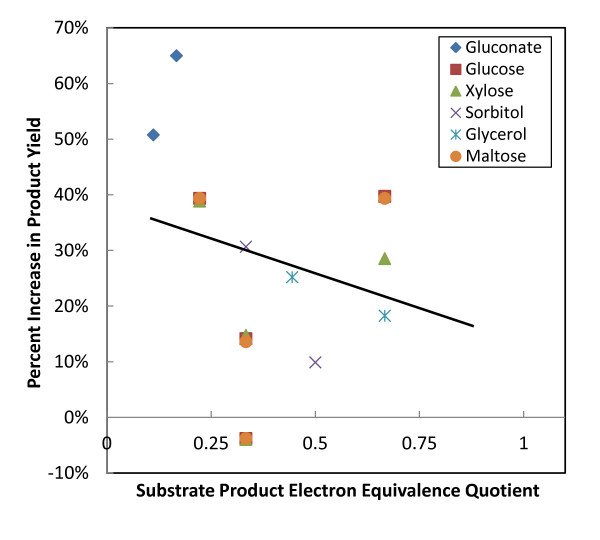
**Theoretical Increases in Product Yield of Growth Coupled Resulting from Electrical Enhancement**. Theoretical improvement in product yield resulting from electrical enhancement for strains with reactions knocked out to produce the desired product.

Figure 4 characterizes the affect that electrical enhancement has on product flux in these strains coupled to biomass production. These results show that in most cases, electrical enhancement is compatible with growth coupled strain designs, and that the improvement in product flux is a function of SPEEQ. As in Section 3.1, there are a few data points that deviate from the general trend. These are associated with carbon fixation. Interestingly, two points for butanol production show a decrease in product yield because bioelectrosynthesis results in increased biomass yield instead as electrons availability enables carbon to be rerouted to form biomass. This result suggests that it is important to recalculate the growth coupled strategies to obtain effective coupling during bioelectrosynthesis.

The yield calculations in Figure 4 are based on substrate uptake rates. We further explored product yield with respect to all incoming carbon (data and figure included in Additional file 5). There was generally no difference in the two different methods for calculating yield since CO_2 _was fixed in only a few scenarios. Where CO_2 _was fixed, the product yield relative to total incoming carbon was slightly lower (<10%) than yield with respect to substrate uptake rate in most cases. An exception was succinate production under electrically enhanced conditions, because the carbon fixing reaction, *ppc*, is part of the succinate producing pathway. The succinate yield relative to total incoming carbon is 21% and 16% for glucose and xylose, respectively. While this is lower than the yield relative to only glucose or xylose, bioelectrosynthesis still improves the overall efficiency of product synthesis since a greater proportion of total incoming carbon ends up in the product.

During biomass growth, NAD^+ ^must be regenerated by reducing partially oxidized metabolic intermediates such as pyruvate to lactate or ethanol (etc.) that are then excreted from the cell. The amount of each intermediate can be modulated by the cell to balance the reducing equivalents consumed and produced during fermentation so that it can grow on a variety of different substrates. However, there exists a balance between managing the redox balance within the cell (consequently directing carbon flow towards these partially oxidized metabolic intermediates, such as ethanol) and maximizing energy yields through other pathways (consequently producing other metabolites that do not consume reducing equivalents but instead produce ATP, such as acetate). Product formation is often coupled to growth via knock-outs that perturb these NAD^+ ^regenerating pathways, forcing carbon flux to be directed towards desired products. Electrical enhancement introduces another redox disturbance to the cell, to direct additional carbon through these pathways. This perturbation can often come at the expense of growth rate.

**Figure 5:**
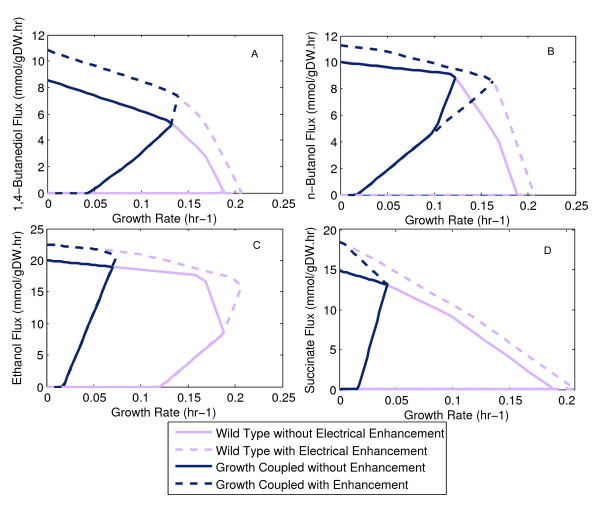
**Production Envelopes for Growth Coupled and Electrically Enhanced Metabolite Production on Glucose**. The production envelopes show changes to growth rate and maximum product flux profiles resulting from electrical enhancement. The trade-off between these suggests that an optima exists both for strain design and for a dynamic strategy.

To understand this better, we examined the production envelopes for individual biochemicals under electrically and non-electrical enhanced conditions with glucose as the substrate. All four graphs in Figure 5 show that electrical enhancement enlarges the production envelope. A larger production envelope means that the possible solutions to coupling product formation to biomass growth may be different, or may lead to different outcomes. Figure 5C clearly shows this for ethanol production. Not only is the new optimum at a higher growth rate but the ethanol flux is much greater. The benefit of improvement in both yield and biomass growth rate is the resulting improvement in overall substrate-specific productivity of these fermentation processes. These results support the conclusions that were obtained in Section 3.2.

However, when comparing these different production envelopes we find that the electrons (or NADH) supplied (generated) by an electrode can, but does not necessarily appear in the desired product. For example, in the case of *n*-butanol production (Figure 5B), electrical enhancement seems to largely impact growth rate without adversely increasing product flux. By comparison, the maximum growth rate for succinate occurs when there is no electrical enhancement and, electrical enhancement increases the succinate yield at the expense of growth rate. In the case of ethanol production, growth rate and product flux increase simultaneously with electron uptake.

This result shows the inherent trade-off that can exist between the improved yield and the growth rate of the organism, but that it appears to be conditional on the active metabolic pathways. Consider further that the optimum electron uptake rate predicted computationally for ethanol production is 20.6 mmol/gDW-hr with a predicted growth rate of 0.075 hr^-1^. By comparison, forcing electrons at 30 mmol/gDW-hr reduces growth rate to 0.058 hr^-1 ^and increases product flux to 21.6 mmol/gDW-hr. This result suggests that the best strategy for electrically enhancing a bioprocess would be a dynamic one. Having no electrical enhancement during the beginning of a batch would maximize growth rate, and once a threshold cell density is reached, growth rate can be sacrificed for improved product yield with electrical enhancement. However, additional optimization will be required to identify the optimal time of switching from a growth phase to a production phase [49]. The implementation of such a dynamic strategy can have a significant impact on substrate specific productivity and the metabolic knockout strategies.

#### 3.4.2 Changes in Flux Distributions for Growth Coupled Products

Under growth coupled product synthesis, fewer reactions are available for the cell to respond to electrically induced redox changes. Inspite of the changes in the metabolism, the network still has to balance the additional reducing equivalents supplied from the electrode. Therefore, the specific pathways or the magnitude of that response may be different from the wild type. We analysed the changes in the flux distribution for the four scenarios in Section 3.4 and a summary of the results are provided in Table 4. Detailed changes to the flux distributions are shown in the Additional files 3. For example, flux through the non-oxidative branch of the TCA cycle is significantly increased at the expense of other pathways such as the pentose phosphate pathway (PPP) and oxidative TCA cycle. Glycolysis is not significantly affected. ATP synthase activity is significantly upregulated to produce ATP as described earlier. While these changes in metabolism are essential to balance the additional reducing equivalent load, the changes do not appear to disrupt growth coupled product formation in most cases except butanol.

**Table 4:** Predicted Changes in Fluxes Through Selected Pathways

	WildType	1,4-Butanediol	Ethanol	*n*-Butanol	Succinate
**Glycolysis**					

Upper (Reaction)	10%	34% (PFK)	-18%	32% (PFK)	-1%

Lower	0%	~0%	0%	-1%	-1%

**Branched TCA**					

Oxidative	10%	4%	-18%	32%	-91%

Reductive	10%	40%	-18%	32%	40%

**Pentose Phosphate Pathway**					

Oxidative	10%	4%	-50%	14%	-91%

Non-Oxidative	0%	4%	>300%	15-20%	-91%

**ATP Synthase**	-216%	246%	146%	-357%	-638%

#### 3.4.3 Large Knock-out Strategy and Productivity

The trade-off between product flux and growth rate is capable of significantly affecting the overall productivity of a system. We examined three specific knock-out strategies for ethanol production to characterize the effect of electrical enhancement on strain design and its productivity.

**Figure 6:**
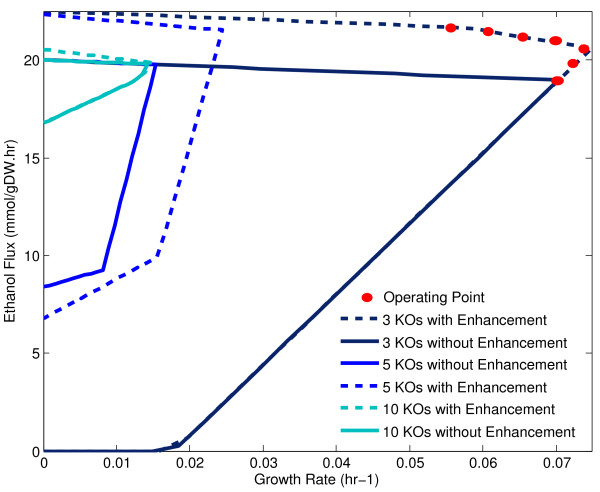
**Production Envelopes for Three Growth Coupled Strategies for Ethanol**. The production envelopes for various growth coupled strategies for ethanol production show the impact that electrical enhancement has on the strategy's maximum product flux and growth rate. These parameters greatly influence overall bioprocess productivity.

The production envelopes for three different strategies are shown in Figure 6 and are based on results obtained by Feist *et al*. [36]. The figure shows that growth coupled product flux is highest for the 10 knockout strategy, followed by the 5 knockout strategy, and finally the 3 knockout strategy. Intuitively, we expect that increasing the number of knockouts results in product fluxes that are closer to the theoretically maximum flux resulting in improved yields. We show, however, that under electrically enhanced conditions, this strategy does not necessarily provide the best product yield. Instead, the 5 knockout strategy and 3 knockout strategy are far superior on a basis of product yield and growth rate. This strongly suggests that while existing strains can be used for bioelectrosynthesis, Opt-Knock or other strain design algorithms such as EMILiO or OptForce that consider electrode reactions will generally lead to different strain designs that are capable of improving product flux.

In addition, since electrical enhancement is essentially current supplied at an electrode, by varying the current, it is possible to operate along a particular section of the production envelope. A number of these operating points represented by blue dots are shown for the 3 knockout strategy. Depending on the magnitude of this current, it is possible to operate along the maximum growth rate, or at some other point that improves the product flux while lowering growth rate. This flexibility has significant consequences for implementing a dynamic strategy that maximizes productivity. Such a strategy would use no current (or low current) early during a batch to maximize biomass and then increase the current to maximize product flux.

## Conclusions

Traditional strategies that attempt to manipulate NADH availability, namely, the utilization of carbon source with different redox states and the genetic manipulation of the host cell are generally well understood. Bioelectrosynthesis is a third method that has been used; however a systematic understanding of the potential of this technique hasn't been fully evaluated. We have provided a systematic understanding of how electrical enhancement can impact cellular metabolism.

Our evaluation of electrical enhancement on a complete genome scale network of the model organism, *E. coli*, shows that electrical generation of reducing equivalents is capable of influencing the three most important aspects of bioprocesses: biochemical yield, cellular ATP yield and cellular growth rate. Understanding these processes has provided insight into the long term strategies for electrical enhancement techniques and we have highlighted the significance of these findings both for the application of electrical enhancement at an industrial scale as well as for rational strain design.

Our results showed that manipulating cellular redox conditions can force the metabolism to increase the utilization of pathways that fix carbon dioxide. For *E. coli *we were able to increase the activity of the phosphoenolpyruvate carboxylase pathway because the increased demand to regenerate NAD^+ ^required the use of this pathway. Since there is significant interest to use bioelectrosynthesis on carbon dioxide only, we extended this concept to compare the difference between sole CO_2 _utilization and co-utilization of CO_2 _and hexose. Our results suggest that while bioelectrosynthesis is possible on CO_2 _alone, its very low yield probably make it unsuitable at an industrial scale unless significant advances are made in understanding and optimizing electron transfer rates from an electrode. A constraint on the upper electron uptake limit means that in the short term at least, electrical enhancement strategies that co-utilize CO_2 _and sugar substrates are more feasible.

The extent to which electrical enhancement is capable of influencing ATP and biomass yields provided a basis for understanding the implications that bioelectrosynthesis has on strain design. Our results suggest that electrical enhancement is generally compatible with existing metabolic engineering strategies, although sometimes it may be necessary to recalculate specific strategies under electrically enhanced conditions. The potential trade-off between the biomass growth rate and product yields suggests that there is room for process optimization during bioelectrosynthesis where the organism may grow under non-electrically conditions initially and then electrically enhanced conditions once certain growth conditions have been met.

Finally additional work needs to be done to understand the microbiology and physiology of electricigens and their ability to accept electrons. There are a number of possible limitations that can arise, and in particular incorporation of components of the electron transport chain from organisms such as *Shewanella*, *Geobacter *or *Acidithiobacillus *to improve electron transfer rates would be the next logical step. Our results suggest that influencing the NADH/NAD^+ ^ratio is possible with an electrode - and in particular that this may form the basis for controlling fluxes through some reactions, notably those that oxidize NADH and alleviate redox constraints. Further work needs to examine the network level adaptations of an organism in response to these redox perturbations using metabolic flux analysis with ^13^C isotope labelled substrates and the scale-up of these systems, in much the same way that fuel cells are being considered for scale up.

This computational study lays the framework for understanding where electrical enhancement is most useful and evaluating potential benefits as well as limitations. The concept of electrical enhancement is promising for products that are highly reduced. These concepts could be beneficial in developing strategies for chemicals or fuels (e.g., jet fuels, biodiesel) that require large amounts of reducing power via NADH. Yields of large molecules such as those that would be required to make fuels could be improved by electrical enhancement.

The experimental validation of the principles described herein is critical to further understand and optimize microbial bioelectrosynthesis of key biochemical products and develop economically viable and environmentally friendly commercial bioprocesses.

## Competing interests

The authors declare that they have no competing interests.

## Authors' contributions

A.V. P. discussed the study, developed the model, performed simulations, interpreted data and wrote the paper. R.M. conceived the study, interpreted the data and wrote the paper. All authors read and approved the final manuscript.

## Supplementary Material

Additional file 1 **Model Reactions/Deletion Strategies**. Click here for file

Additional file 2 **Flux Profiles - Maximizing Biomass; CO_2 _Incorporating Pathways, Maximizing ATP**. Click here for file

Additional file 3 **Metabolic Maps of Changes in Flux Distributions**. Click here for file

Additional file 4 **Maximizing Product Flux Sample of Glucose Flux Profile**. Click here for file

Additional file 5 **Changes in Flux Distribution; Yield Data Based on Total Incoming Carbon**. Click here for file
