# Supplementary figures and images for: In silico characterization of microbial electrosynthesis for metabolic engineering of biochemicals
Source: Microb Cell Fact. 2011 Oct 3;10:76. doi: 10.1186/1475-2859-10-76 (PMC3215969; doi:10.1186/1475-2859-10-76)

# Ethanol

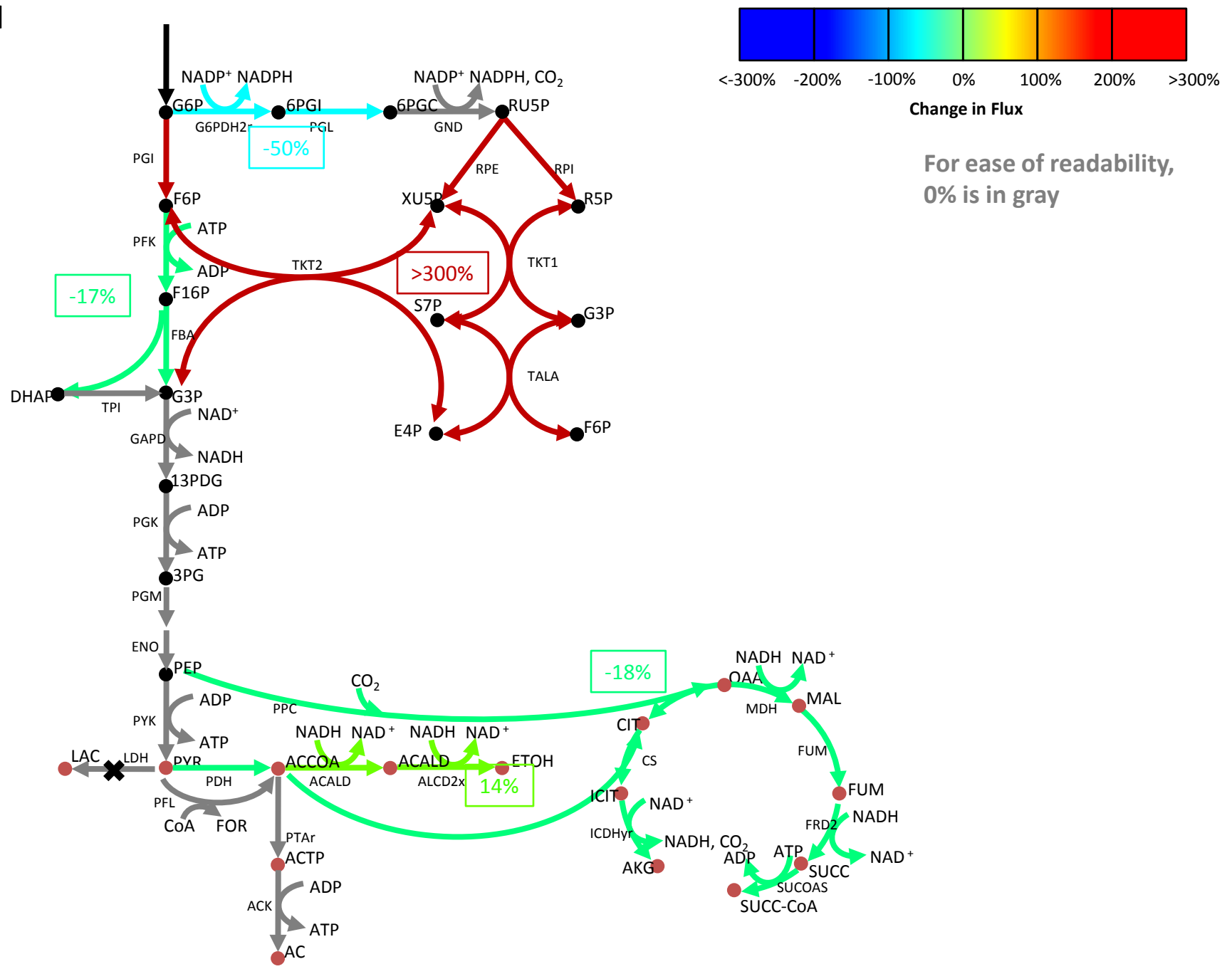

# Succinate

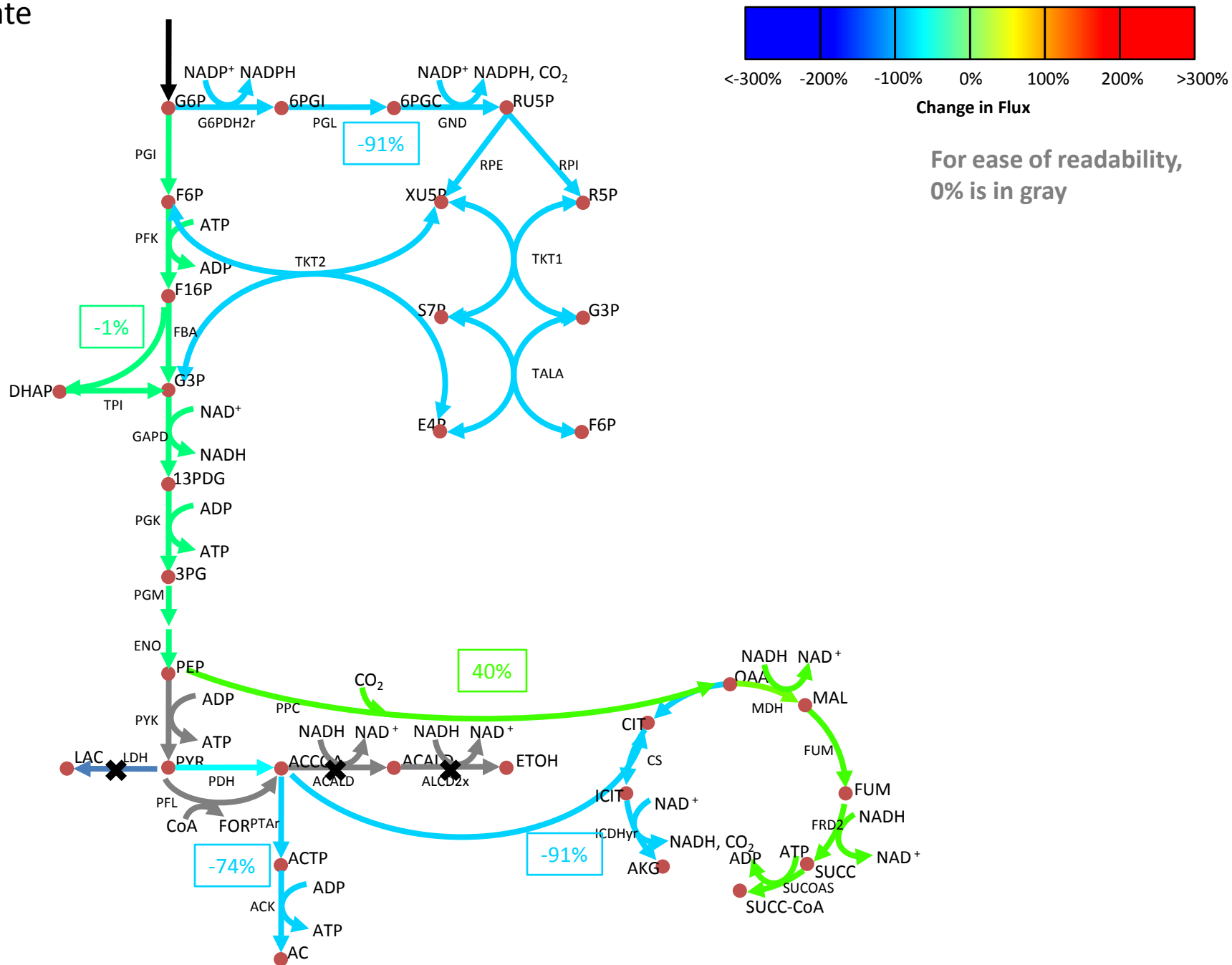

Butanol

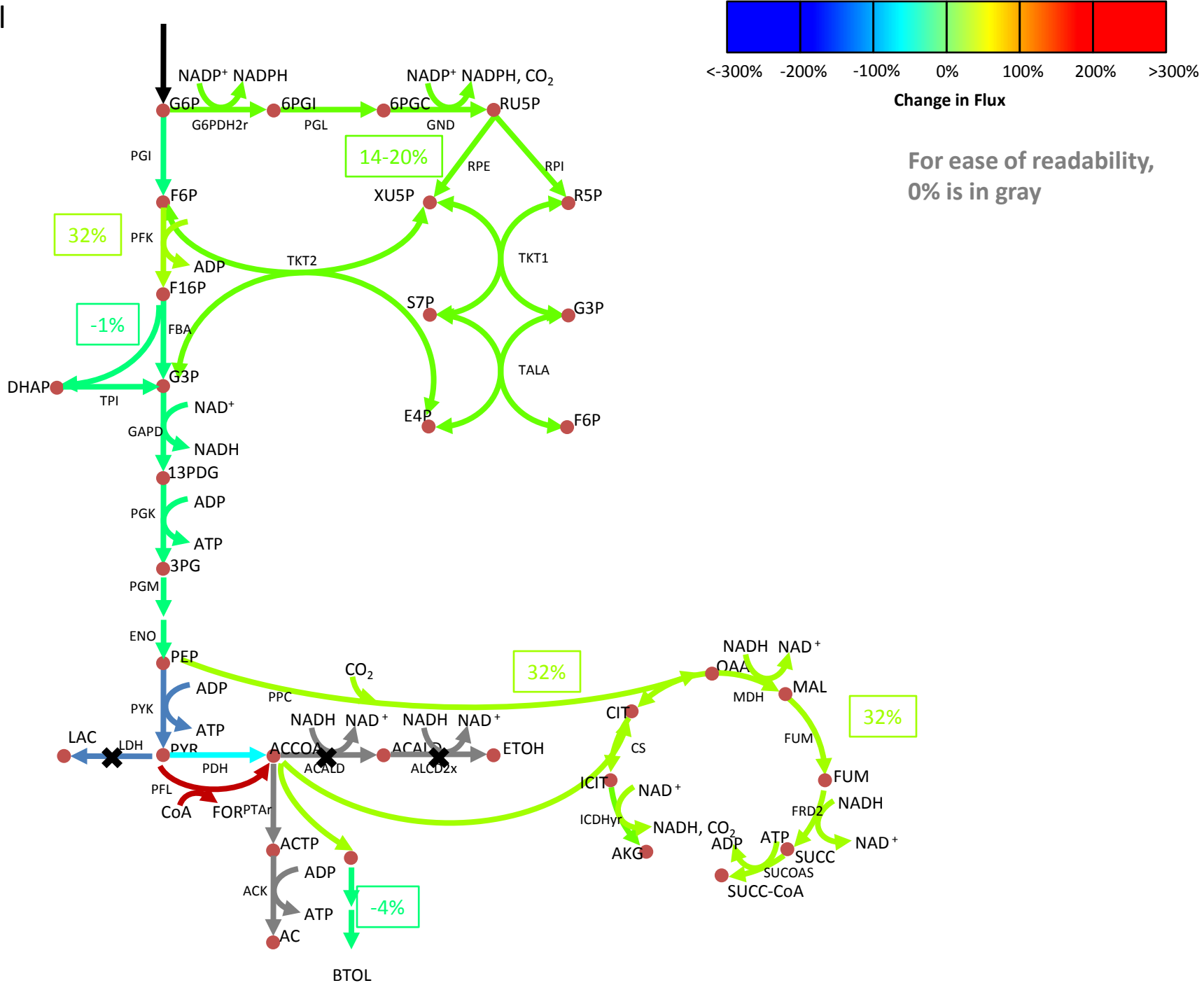

# Butanediol

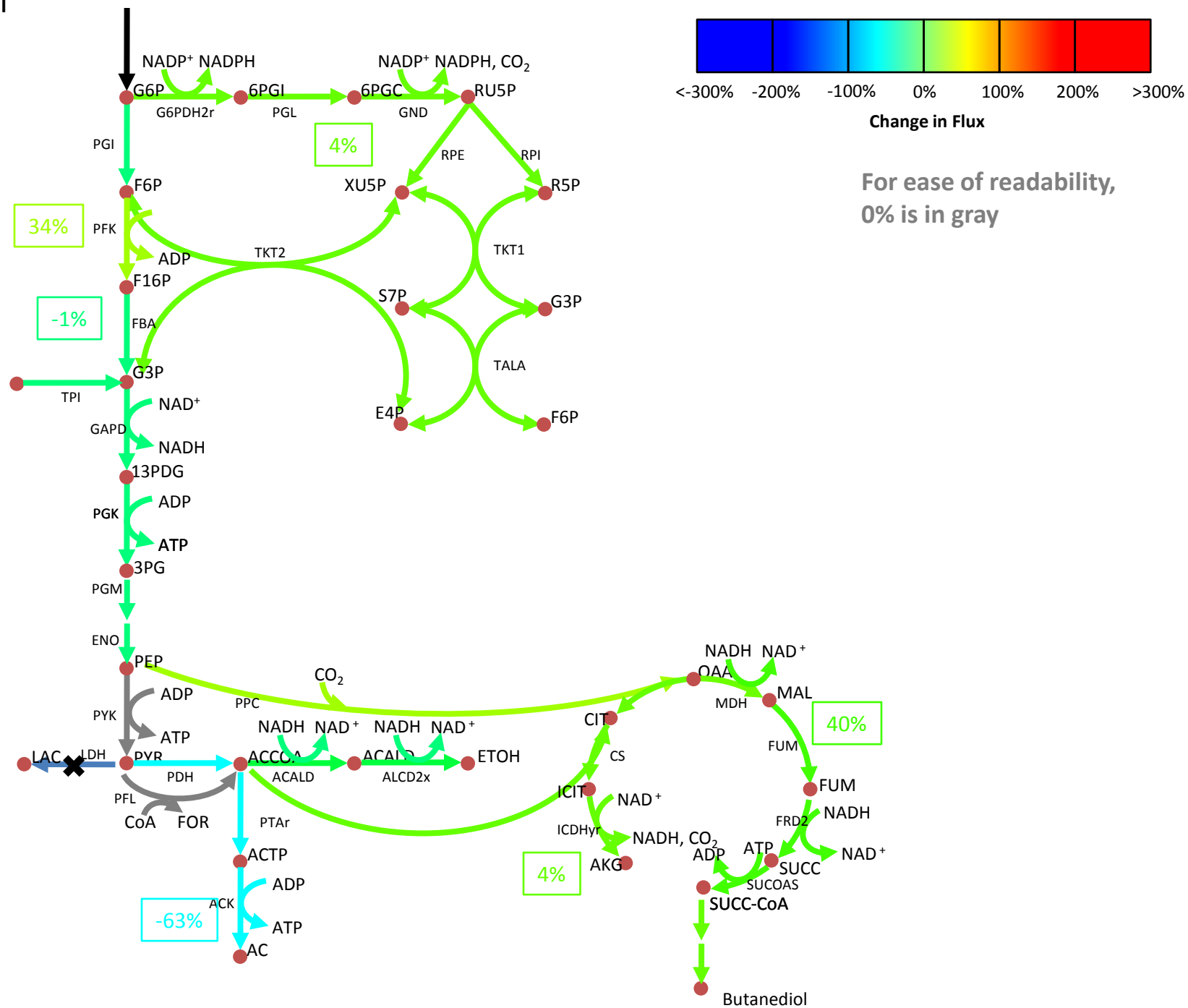

## Wild Type

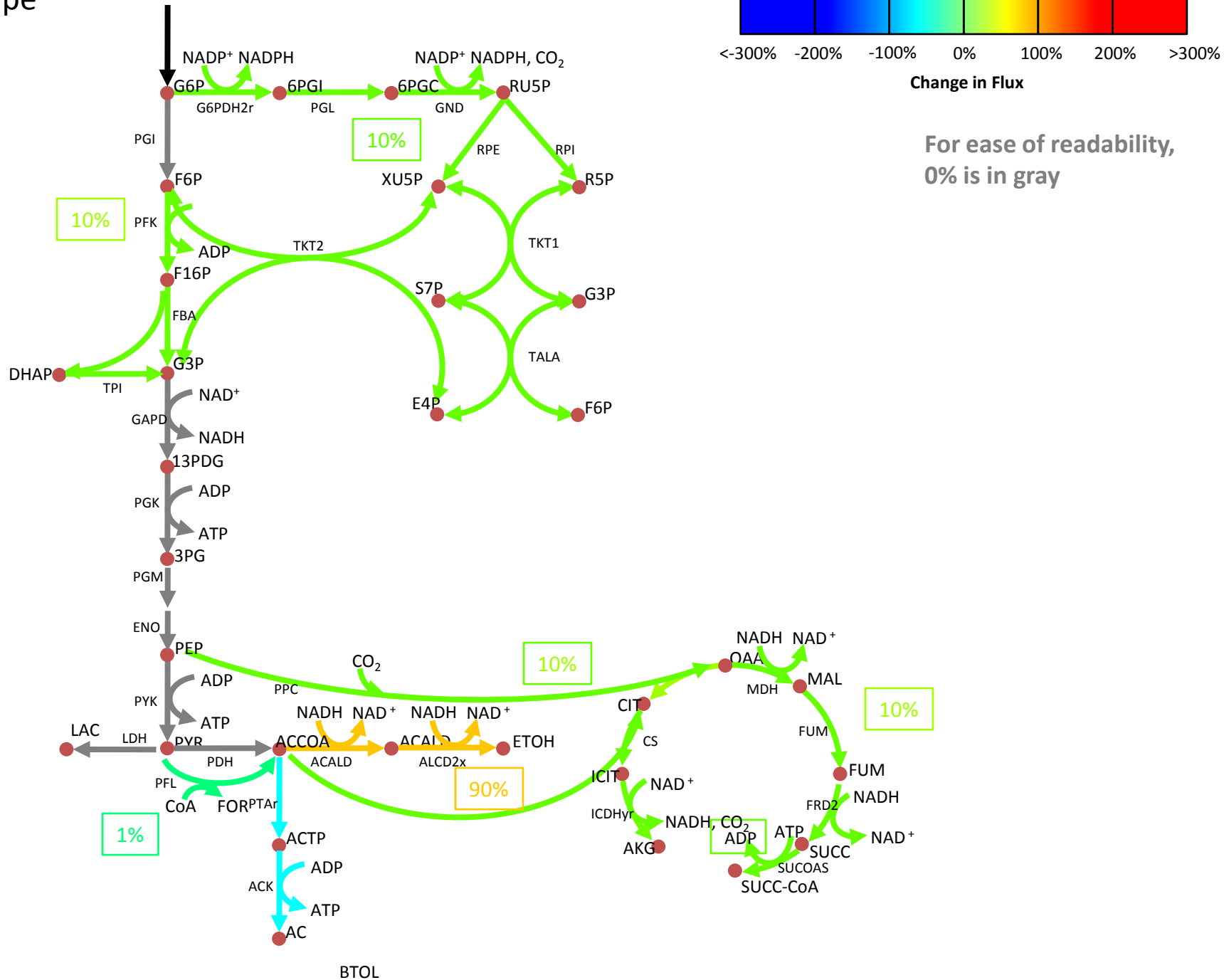

Supplement: Additional file 3 — Metabolic Maps of Changes in Flux Distributions. [file 1475-2859-10-76-S3.PDF]
